# Sex- and age-dependent association of *SLC11A1 *polymorphisms with tuberculosis in Chinese: a case control study

**DOI:** 10.1186/1471-2334-7-19

**Published:** 2007-03-19

**Authors:** Kim Hung Leung, Shea Ping Yip, Wa Sang Wong, Lap San Yiu, Kam Keung Chan, Wai Man Lai, Eudora YD Chow, Che Kit Lin, Wing Cheong Yam, Kin Sang Chan

**Affiliations:** 1 Department of Health Technology and Informatics, The Hong Kong Polytechnic University, Hong Kong SAR, China; 2 TB Laboratory, Haven of Hope Hospital, Hong Kong SAR, China; 3 Department of Pulmonary and Palliative Care, Haven of Hope Hospital, Hong Kong SAR, China; 4 Department of Pathology, United Christian Hospital, Hong Kong SAR, China; 5 Hong Kong Red Cross Blood Transfusion Service, Hong Kong SAR, China; 6 Department of Microbiology, The University of Hong Kong, Hong Kong SAR, China; 7 Department of Microbiology, The Chinese University of Hong Kong, Hong Kong SAR, China

## Abstract

**Background:**

Host genetic factors are important determinants in tuberculosis (TB). The *SLC11A1 *(or *NRAMP1*) gene has been studied extensively for genetic association with TB, but with inconsistent findings. In addition, no study has yet looked into the effect of sex and age on the relationship between *SLC11A1 *polymorphisms and TB.

**Methods:**

A case-control study was conducted. In total, 278 pulmonary TB patients and 282 sex- and age-matched controls without TB were recruited. All subjects were ethnic Chinese. On the basis of linkage disequilibrium pattern, three genetic markers from *SLC11A1 *and one from the nearby *IL8RB *locus were selected and examined for association with TB susceptibility. These markers were genotyped using single strand conformation polymorphism analysis or fragment analysis of amplified products.

**Results:**

Statistically significant differences in allele (*P *= 0.0165, OR = 1.51) and genotype (*P *= 0.0163, OR = 1.59) frequencies of the linked markers SLC6a/b (classically called D543N and 3'UTR) of the *SLC11A1 *locus were found between patients and controls. With stratification by sex, positive associations were identified in the female group for both allele (*P *= 0.0049, OR = 2.54) and genotype (*P *= 0.0075, OR = 2.74) frequencies. With stratification by age, positive associations were demonstrated in the young age group (age ≤65 years) for both allele (*P *= 0.0047, OR = 2.52) and genotype (*P *= 0.0031, OR = 2.92) frequencies. All positive findings remained significant even after correction for multiple comparisons. No significant differences were noted in either the male group or the older age group. No significant differences were found for the other markers (one *SLC11A1 *marker and one *IL8RB *marker) either.

**Conclusion:**

This study confirmed the association between *SLC11A1 *and TB susceptibility and demonstrated for the first time that the association was restricted to females and the young age group.

## Background

Tuberculosis (TB) remains the worldwide leading cause of morbidity and mortality due to the infection with *Mycobacterium tuberculosis *(MTB). This successful infectious agent kills about three million people annually and has been estimated to infect one-third of the world population [1]. The global emergence of TB is due to the pandemic of acquired immunodeficiency disease syndrome and the development of multidrug resistant strains of MTB [2]. In Hong Kong, TB remains prevalent and is still a leading cause of death due to infectious diseases in the past decade [3,4].

Only 10% of individuals infected with MTB develop clinical disease. There are many known factors affecting TB development, such as age, poverty, sex, alcohol, malnutrition, diabetes and human immunodeficiency virus infection [5-9]. Host genetic factors are also important determinants, as are evident from the different concordance rates in monozygotic and dizygotic twins and the racial difference in susceptibility to MTB infection [10,11]. Of the TB susceptibility genes identified so far, the solute carrier family 11, member 1 (*SLC11A1*) gene is the one most extensively studied. The mouse homolog *Slc11a1 *was first cloned as the *Ity/Lsh/Bcg *locus [12]. A point mutation in this gene resulted in increased susceptibility in mouse to infections with *S. typhimurium*, *L. donovani *and *M. bovis *[13-16].

The human *SLC11A1 *gene, also known as *NRAMP1*, is located on chromosome 2q35 and has 15 exons spanning about 14 kb [17]. The gene encodes a transmembrane protein expressed exclusively in macrophages/monocytes and polymorphonuclear leukocytes [18,19]. The protein is strongly believed to act as a transporter of divalent cations, in particular iron ions (Fe^2+^), across the phagosomal membrane [20-22]. The regulation of Fe^2+ ^level in the microenvironment of the phagosome is important for the control of MTB. Since iron is essential for biological systems, both the human host and the bacterium compete for iron in favour of effective immunity and establishing infection, respectively, during infection [23,24]. Hence, an optimal iron status of the host has to be maintained in order to limit the availability of this essential nutrient to the bacteria, but supply it sufficiently to the host defensive cells for the generation of reactive intermediates of oxygen and nitrogen [23]. In addition, SLC11A1 has pleiotropic effects on the activation of macrophages, which play a critical role in innate immunity against MTB [24].

The relationship between *SLC11A1 *polymorphisms and TB has been extensively studied in many different populations since the first report by Bellamy and co-workers [25]. Although a recent meta-analysis systematically reviewed and summarized the data published in the last ten years [26], inconsistencies are common among different studies. In addition, sex and age of cases and controls were usually not well matched in most published studies. Therefore, no study has yet looked into the effect of sex and age on the relationship between *SLC11A1 *polymorphisms and TB. The present study recruited cases and controls well matched for both factors and reported for the first time the effect of these factors on the relationship.

Our recent systematic study of the linkage disequilibrium (LD) pattern of the *SLC11A1 *locus demands that association studies involving *SLC11A1 *be carried out using genetic markers from its 5' end like SLC1 and also from its 3' end like SLC6a and SLC6b [27]. True positive association results indicate either the direct effect of the *SLC11A1 *gene itself on the disease of interest or some other nearby genes because of the LD phenomenon [28]. Our LD study also indicates that a marker (IL8rb) in the *IL8RB *locus at least 220 kb upstream of *SLC11A1 *is in significant and strong LD with 5' *SLC11A1 *markers [27]. The *IL8RB *gene encodes one of the receptors for interleukin-8 and is expressed in neutrophils. This receptor plays a role in the immune response in infectious diseases and in tuberculosis [29-32]. In view of the LD relationship and the biological functions of the *IL8RB *gene, our LD study also recommends that the *IL8RB *locus be tested for association with TB [27]. Therefore, four markers were tested for association with TB in the present study: SLC1 (alleles (GT)_9_, (GT)_10 _and (GT)_11_), SLC6a (alleles G and A), SLC6b (alleles TGTG and ----) and IL8rb (alleles C and T) (shown in nomenclature used in our LD study). Other notations that have been used for these markers include the following: 5'(GT)_n _or 5'(CA)_n _for SLC1; D543N or rs17235409 for SLC6a; 1729+55del4, 3'UTR or rs17235416 for SLC6b; and CXCR2-786C/T or rs230054 for IL8rb. Since SLC6a and SLC6b are in perfect LD in Chinese population [27], they are regarded as a single combined marker SLC6a/b (alleles G-TGTG and A- ----) in the present study.

## Methods

### Patients and control subjects

A total of 278 blood samples from unrelated patients with pulmonary TB (TBP) were collected from three different local hospitals. All these case subjects were ethnic Chinese patients with proven pulmonary TB diagnosed by positive culture results of mainly sputum and very occasionally bronchial aspirates or washings (with or without positive acid-fast smear results). Positive culture results were also confirmed by a nucleic acid-based assay: AccuProbe *Mycobacterium tuberculosis *Complex Culture Identification Test (Gen-Probe, San Diego, CA, USA) in Haven of Hope Hospital, LCx *Mycobacterium tuberculosis *Assay (Abbott Laboratories; Abbot Park, Ill, USA) in United Christian Hospital, or an in-house nested PCR assay [33-35] in Queen Mary Hospital. Bacteriological and molecular tests were carried out by the microbiology laboratories of the respective collaborating hospitals. Blood samples were also collected from 282 unrelated Chinese individuals who were matched with the cases for sex and age, and were used as control subjects (TBC). Among the controls, 236 samples were from hospital patients without active pulmonary TB who had negative acid-fast smear results and did not return for medical consultation for respiratory symptoms within the 3-year period of subject recruitment. Forty-six samples were from healthy blood donors. The majority of samples (85% of cases and 84% of controls) were collected from Haven of Hope Hospital. The study was approved by local institutional review boards. DNA was extracted from whole blood samples by a modified salting-out method [36] or by using a Qiagen Blood Kit (Qiagen, CA, USA).

### PCR amplification and genotyping of genetic markers

Three fragments were amplified by polymerase chain reaction for the genotyping of four genetic markers SLC1, SLC6a, SLC6b and IL8rb, as described in our previous LD study [27]. Briefly, markers SLC6a and SLC6b were on the same PCR fragment because of their physical proximity. SLC6a/b and IL8rb were genotyped by the technique of single strand conformation polymorphism. SLC1 is a microsatellite marker and was genotyped by both fragment analysis on an ABI PRISM 310 Genetic Analyzer (Applied Biosystems, Foster City, USA) and restriction enzyme digestion with *Rsa*I (MBI Fermentas, Vilnius, Lithuania).

### Statistical analysis

Distribution of genotypes was assessed for Hardy-Weinberg equilibrium by χ^2 ^test or exact test if assumptions for χ^2 ^distribution was violated. Allele and genotype frequencies of cases and controls were compared using χ^2 ^test to investigate the association between genetic markers and TB. Odds ratios (OR) with 95% confidence intervals (95% CI) were also calculated to show the effect size. Correction for multiple comparisons between cases and controls was carried out using false discovery rate (FDR) [37]. The FDR is the expected proportion of true null hypotheses rejected out of the total number of null hypotheses rejected. The procedure of controlling the FDR at a level of 0.05 was performed as described by Benjamini and co-workers [38].

## Results

### Demographics

In this case-control study, 278 TB patients (TBP) and 282 sex- and age-matched controls (TBC) without TB were recruited. Both groups had the same proportion of each sex (74% male). Both groups also had the same mean age (65 years old) with very similar standard deviations (18.4 years for TBP and 18.2 years for TBC) and age ranges (14 – 98 years for TBP and 17 – 99 years for TBC).

### Relation between *SLC11A1 *and *IL8RB *polymorphisms and tuberculosis

The distribution of allele and genotype frequencies of the markers tested is summarized in Table 1. The genotypes of all polymorphisms in both the TBP and TBC groups were in Hardy-Weinberg proportions. Markers SLC1 of *SLC11A1 *and IL8rb of *IL8RB *did not show any significant differences in allele and genotype frequencies between cases and controls.

**Table 1:** Relation between *SLC11A1 *and *IL8R *polymorphisms and tuberculosis in Chinese

**Alleles/genotypes**	**TB patients**		**Control subjects**		**Odds ratio (95% CI)**	***P *value**
**Comparison of allele frequencies**						
**SLC1^a^**						
(GT)_9_	478	(86%)	493	(87%)	1	
Others	78	(14%)	71	(13%)	1.13 (0.80 – 1.60)	0.4780
**SLC6a/b^b^**						
G-TGTG	462	(83%)	497	(88%)	1	
A- ----	94	(17%)	67	(12%)	**1.51 (1.08 – 2.12)**	**0.0165^c^**
**IL8rb**						
C	370	(67%)	395	(70%)	1	
T	186	(33%)	169	(30%)	1.18 (0.91 – 1.51)	0.2096
						
**Comparison of genotype frequencies**						
**SLC1^a^**						
(GT)_9_/(GT)_9_	204	(73%)	214	(76%)	1	
(GT)_9_/others	70		65			
		(27%)		(24%)	1.14 (1.09 – 2.32)	0.4957
others/others	4		3			
**SLC6a/b^b^**						
G-TGTG/G-TGTG	192	(69%)	220	(78%)	1	
G-TGTG/A- ----	78		57			
		(31%)		(22%)	**1.59 (1.09 – 2.32)**	**0.0163^c^**
A- ----/A- ----	8		5			
**IL8rb**						
C/C	122	(44%)	135	(48%)	1	
C/T	126		125			
		(56%)		(52%)	1.17 (0.84 – 1.64)	0.3438
T/T	30		22			

^a ^"Others" represent the minor alleles (GT)_10 _and (GT)_11 _of the marker SLC1.

^b ^Markers SLC6a and SLC6b are in perfect LD and thus are analyzed as a single marker (SLC6a/b).

^c ^Significant even after correction of multiple testing based on false discovery rate at a level of 0.05. The adjusted cut-off *P *value is 0.0167 for six comparisons. For details, see additional file 1.

On the other hand, the single combined marker SLC6a/b showed significant differences between TBP and TBC (Table 1) in the allele frequencies (χ^2 ^= 5.747, *P *= 0.0165) and in the genotype frequencies (χ^2 ^= 5.767, *P *= 0.0163). With reference to the major allele G-TGTG, the odds ratio (OR) for the minor allele A- ---- was 1.51 (95% confidence intervals [CI], 1.08 – 2.12). With reference to the common homozygous genotype G-TGTG/G-TGTG, genotypes with at least one copy of the allele A- ---- had a combined OR of 1.59 (95% CI, 1.09 – 2.32). Since genotypes G-TGTG/A- ---- and A- ----/A- ---- gave similar ORs (1.57 and 1.83) and homozygotes A- ----/A- ---- were only found in small numbers in both groups, the combined OR of 1.59 was determined. It is important to note that the differences in allele and genotype frequencies remained significant even after correction for multiple comparisons (n = 6) by false discovery rate (FDR) [37] at a level of 0.05 (See additional file 1).

### Stratified analysis for the marker SLC6a/b

The frequency data of the SLC6a/b marker were further analyzed with stratification of the subjects by sex *or *age (Table 2). When the subjects were stratified by sex, significant differences between TBP and TBC were only observed in the female group for both allele frequencies (*P *= 0.0049, OR = 2.54) and genotype frequencies (*P *= 0.0075, OR = 2.74). When the subjects were stratified by age into two groups (≤65 years or > 65 years), significant differences between TBP and TBC were only found in the younger age group, also for both allele frequencies (*P *= 0.0047, OR = 2.52) and genotype frequencies (*P *= 0.0031, OR = 2.92). No significant differences were noted in either the male group or the older age group. The differences between TBP and TBC in the female group and the younger age group still remained significant after correction for multiple testing (n = 8) by FDR at a level of 0.05 (See additional file 1). It was worth noting that the mean age (in years) remained essentially the same with or without stratification by sex: 65.4 for male TBP and male TBC, 63.7 for female TBP, 63.8 for female TBC, and 65.0 for the overall TBP and the overall TBC. It was also true that the proportions of male subjects remained essentially the same with or without stratification by age: 74% except for the old TBP (75%). With stratification by age, the mean age (in years) was 46.9 for young TBP, 47.0 for young TBC, 77.7 for old TBP and 77.6 for old TBC.

**Table 2:** Relation between markers SLC6a/b and tuberculosis in Chinese stratified by sex *or *age^a^

**Alleles/genotypes**	**TB patients**		**Control subjects**		**Odds ratio (95% CI)**	***P *value**
**Comparison of allele frequencies**						
***Stratification by sex***						
**Male**						
G-TGTG	352	(85%)	366	(88%)	1	
A- ----	62	(15%)	52	(12%)	1.24 (0.83 – 1.84)	0.2876
**Female**						
G-TGTG	110	(78%)	131	(90%)	1	
A- ----	32	(22%)	15	(10%)	**2.54 (1.31 – 4.93)**	**0.0049^b^**
***Stratification by age***						
**Age ≤65**						
G-TGTG	198	(86%)	218	(94%)	1	
A- ----	32	(14%)	14	(6%)	**2.52 (1.31 – 4.85)**	**0.0047^b^**
**Age >65**						
G-TGTG	264	(81%)	279	(84%)	1	
A- ----	62	(19%)	53	(16%)	1.24 (0.83 – 1.85)	0.3023
						
**Comparison of genotype frequencies^c^**						
***Stratification by sex***						
**Male**						
G-TGTG/G-TGTG	149	(72%)	161	(77%)	1	
A- ----/-	58	(28%)	48	(23%)	1.31 (0.84 – 2.03)	0.2370
**Female**						
G-TGTG/G-TGTG	43	(61%)	59	(81%)	1	
A- ----/-	28	(39%)	14	(19%)	**2.74 (1.29 – 5.82)**	**0.0075^b^**
***Stratification by age***						
**Age ≤65**						
G-TGTG/G-TGTG	86	(75%)	104	(90%)	1	
A- ----/-	29	(25%)	12	(10%)	**2.92 (1.41 – 6.07)**	**0.0031^b^**
**Age >65**						
G-TGTG/G-TGTG	106	(65%)	116	(70%)	1	
A- ----/-	57	(35%)	50	(30%)	1.25 (0.79 – 1.989)	0.3479

^a ^Markers SLC6a and SLC6b are in perfect LD and thus are analyzed as a single marker (SLC6a/b).

^b ^Significant even after correction of multiple testing based on false discovery rate at a level of 0.05. The adjusted cut-off *P *value is 0.0250 for eight comparisons. For details, see additional file 1.

^c ^The genotype A- ----/- stands for either A- ----/G-TGTG or A- ----/A- ----.

When the subjects were stratified by both sex *and *age, no significant differences between TBP and TBC were observed among different sex-age groups after correction for multiple testing by FDR (Table 3).

**Table 3:** Relation between markers SLC6a/b and tuberculosis in Chinese stratified by sex *and *age^a^

**Alleles/genotypes**	**TB patients**		**Control subjects**		**Odds ratio (95% CI)**	***P *value**
**Comparison of allele frequencies**						
***Stratification by sex and age***						
**Male & age ≤65**						
G-TGTG	151	(89%)	163	(95%)	1	
A- ----	19	(11%)	9	(5%)	2.28 (1.00 – 5.13)	0.0450^b^
**Female & age ≤65**						
G-TGTG	47	(78%)	55	(92%)	1	
A- ----	13	(22%)	5	(8%)	3.04 (1.01 – 9.16)	0.0408^b^
**Male & age >65**						
G-TGTG	201	(82%)	203	(83%)	1	
A- ----	43	(18%)	43	(17%)	1.01 (0.63 – 1.61)	0.9668
**Female & age >65**						
G-TGTG	63	(77%)	76	(88%)	1	
A- ----	19	(23%)	10	(12%)	2.29 (0.99 – 5.29)	0.0478^b^
						
**Comparison of genotype frequencies^c^**						
***Stratification by sex and age***						
**Male & age ≤65**						
G-TGTG/G-TGTG	67	(79%)	79	(92%)	1	
A- ----/-	18	(21%)	7	(8%)	3.03 (1.19 – 7.70)	0.0158^b^
**Female & age ≤65**						
G-TGTG/G-TGTG	19	(63%)	25	(83%)	1	
A- ----/-	11	(37%)	5	(17%)	2.90 (0.86 – 9.75)	0.0798
**Male & age >65**						
G-TGTG/G-TGTG	82	(67%)	82	(67%)	1	
A- ----/-	40	(33%)	41	(33%)	0.98 (0.57 – 1.66)	0.9276
**Female & age >65**						
G-TGTG/G-TGTG	24	(59%)	34	(67%)	1	
A- ----/-	17	(41%)	17	(33%)	2.68 (1.02 – 7.00)	0.0419^b^

^a ^Markers SLC6a and SLC6b are in perfect LD and thus are analyzed as a single marker (SLC6a/b).

^b ^*Not *significant after correction of multiple testing based on false discovery rate at a level of 0.05. For details, see additional file 1.

^c ^The genotype A- ----/- stands for either A- ----/G-TGTG or A- ----/A- ----.

## Discussion

The present study was a population-based genetic association study in which both cases and controls were unrelated ethnic Chinese in Hong Kong. That both case and control subjects were unrelated individuals from the same ethnic group helps avoid false results due to population stratification, which of course can also be circumvented by family-based association studies [39]. Cases were patients with active pulmonary TB diagnosed by positive culture results and a molecular assay. Culture is much more sensitive than direct microscopy although a few studies reporting the association of *SLC11A1 *polymorphisms and TB relied only on positive direct smears to identify TB cases [25,40,41]. For the control subjects recruited in the present study, the majority were hospital patients who were tested negative for direct smears when first recruited and were not diagnosed to suffer from pulmonary TB within the 3-year period of subject recruitment. The use of hospital patients without the disease under study as control subjects in case-control studies is well documented [42,43]. In fact, two association studies of *SLC11A1 *polymorphisms also recruited hospital patients as controls [41,44].

As the prevalence of tuberculosis varies with sex and age [5,6], they are potential confounding factors in case-control studies of tuberculosis. The present study controlled these confounders by matching cases and controls for these two factors: both cases and controls had the same mean age (65 years old) and the same proportions of both sexes (74% male). As has been reviewed recently [26], cases and controls were usually not well matched for gender and age in previous studies except for a few studies [45,46]. For example, the proportion of male patients was 67% while the controls were all male blood donors in one study [25]. The difference in mean age between cases and controls could be as large as 19.8 years [47]. In addition, the mean age could range from 19 years to 58 years for cases, and from 27 years to 61 years for controls in different studies [26].

Both markers SLC1 and IL8rb did not show any statistically significant difference in allele and genotype frequencies between TBP and TBC in the Chinese population under study (Table 1). This is consistent with the finding that IL8rb was found to show strong LD with 5' *SLC11A1 *markers (like SLC1) but not with 3' *SLC11A1 *markers (SLC6a and SLC6b) [27]. The negative finding with IL8rb of the *IL8RB *locus is supported by a recent American study conducted for white Americans and African Americans [48]. On the other hand, the negative finding with SLC1 seems to contradict current evidence. Meta-analysis of seven studies gave a summary allelic OR of 1.32 (95% CI, 1.03 – 1.68; *P *= 0.026) for SLC1 (also called 5'(GT)_n _or 5'(CA)_n_) [26]. Among these seven studies which included only one study involving Asians (Japanese), between-study heterogeneity was found for ethnicity, mean age of cases and study size. In addition, an intriguing observation is worthy of attention. Among *all *the studies reviewed by the meta-analysis, there were six case-control studies of *SLC11A1 *that involved Asian populations (Chinese, Japanese, Korean and Indian) but did not examine the marker SLC1. One Japanese study did not examine SLC1 because SLC1 was in perfect LD with INT4 (or SLC3 in the nomenclature of our LD study) [49]. This study did not find any association between SLC3 and TB, and this implies that SLC1 was not associated with TB either in this study. One Chinese study found no association between SLC3 and TB susceptibility [45] and this tends to suggest a negative finding with SLC1 in the light of strong LD between SLC1 and SLC3 in Chinese population [27]. One Indian study revealed in the discussion section that the investigators did not publish the negative result of association for SLC1 [50]. The reason for examining other *SLC11A1 *markers (SLC6a and SLC6b) but not SLC1 were not stated in the other three studies [51-53]. It is possible that these authors might have chosen not to report their negative results. In summary, there is only one report of positive association between SLC1 and TB susceptibility in Asian populations. On the contrary, association between SLC1 and TB susceptibility is more consistent in African populations [26]. This observation raises the possibility of genetic heterogeneity of TB susceptibility with respect to this particular microsatellite marker although differences in sample size and how TB is diagnosed can also produce discrepancies among different studies. It should also be noted that, in African populations where both 3' and 5' polymorphisms of the *SLC11A1 *locus were found associated with TB susceptibility, the 3' and 5' polymorphisms in fact contributed *separate *and *independent *main effects [25,54].

Although SLC3 (or INT4) was not tested in the present study because of its strong LD with SLC1, it is expected to give negative finding as SLC1. Meta-analysis of six studies indeed indicates a negative finding with a summary allelic OR of 1.14 (95% CI, 0.96 – 1.35; *P *= 0.13) [26]. In fact, only one of these six studies gave a positive association result.

With correction for multiple testing by FDR, our study showed statistically significant differences in allele and genotype frequencies of the marker SLC6a/b (i.e., D543N and 3'UTR) of the *SLC11A1 *gene between TB patients and sex- and age-matched control individuals (Table 1). The present study suggested that the allele A- ---- acted in a *dominant *fashion to increase the risk of contracting TB because the ORs were very similar for both allelic and genotypic comparisons (1.51 and 1.59 respectively), and the ORs were also similar with either one or two copies of the allele. However, this can only be regarded as tentative because the less common homozygotes (A- ----/A- ----) were found in too small numbers to draw reliable conclusions – a point common to all reported studies. Note that the minor alleles of both SLC6a and SLC6b are much less common in Caucasians than in non-Caucasians [27]. It is possible that this difference in allele frequency might partly explain the racial difference in TB susceptibility. The recently published meta-analysis revealed two important points [26]. First, meta-analysis of ten studies gave a summary allelic OR of 1.33 (95% CI, 1.08 – 1.63; *P *= 0.008) for SLC6b by random effects. Meta-analysis of nine studies gave a summary allelic OR of 1.67 (95% CI, 1.35 – 2.05; *P *< 0.001) for SLC6a by fixed effects. In other words, both markers are associated with susceptibility to TB. Interestingly, subgroup analysis indicated consistent positive association results for both markers for Asian populations only, but not for European and African populations. Second, different studies always gave consistent results for these two markers, either both positive or both negative.

In the present study, the cases and the controls were matched for both sex and age, and had a very wide age range (from <20 to about 100 years old). This allowed the effect of the polymorphisms to be investigated in different strata. For stratification by age, the cut-off point of 65 years was used in this study as it was the mean age of the recruited patients and controls. This cut-off point thus allowed similar sample sizes in both young and old age groups. In addition, the incidence of tuberculosis tended to increase between 60 and 70 years of age for the patients [55-57]. It would thus be interesting to compare the effect of the polymorphisms in the group with lower incidence and the group with higher incidence. Indeed, differences in allele and genotype frequencies were found significant only in the female group and the younger age group (age ≤65 years), but not in the male group or the older age group (Table 2). These differences remained significant even after correction of multiple comparisons by FDR at a level of 0.05 (See additional file 1). Comparisons were also tested with stratification by both sex *and *age, and no significant result was observed. In this case, the numbers of subjects in the cells of the contingency tables probably became too small to give enough power for the stratified analysis (Table 3). The results indicated that the SLC6a/b polymorphisms of *SLC11A1 *contributed to TB development in females or those 65 years old or younger. These findings suggested the importance of sex- and age-matching in both cases and controls since both factors influenced the results in association study. This might explain some of the inconsistent results in association studies between *SLC11A1 *and TB. Note that sex- and age-dependent association between *SLC11A1 *and TB has not been reported before. We recommend that this sex- and age-dependent association be replicated using independent sample sets from different populations.

Globally, the prevalence of TB is similar in males and females until adolescence, and higher in males afterwards [6]. The ratio of female to male TB cases is 1:2 although the reasons for this difference are unclear. However, women were reported to be at increased risk of TB during their reproductive years [6]. In Hong Kong, the prevalence of TB was consistently higher in men than in women in the past five decades [58]. Well-developed health organizations monitor and provide health care to both sexes, and under-notification seems unlikely in Hong Kong. Thus, biological differences probably account for most of the difference in TB rate between males and females. Interestingly, only females showed the significant association between SLC6a/b and TB in the present study. This finding remains to be explained in biological terms. It is also interesting to note that, due to menstruation, females tend to have a lower body iron stores [59-61]. This might lead to a limited supply of iron ions in phagosomes for generating enough hydroxyl radical for bactericidal activity.

The elderly are a highly susceptible group with a higher case rate of TB [55,56]. This is due to the presence of other underlying chronic diseases and the biological changes associated with ageing. More importantly, the immune response declines with ageing and hence is impaired in the microbial clearance mechanisms [62]. As a result, the elderly are at a higher risk of TB development, which is more likely due to the ageing effect rather than direct genetic effects. On the other hand, young adults have stronger immune system in defence against MTB so that they are less likely to develop the disease. Therefore, disease development in young patients is more likely due to factors other than age. This might explain the positive association of the *SLC11A1 *markers SLC6a/b in young patients rather than in elderly patients with TB. The finding suggests that *SLC11A1 *as a TB susceptibility gene plays a significant role in young rather than old patients with TB.

## Conclusion

In conclusion, the present study showed the association of the polymorphisms SLC6a (D543N) and SLC6b (3'UTR) of the *SLC11A1 *locus with TB and supported previous findings [26]. In addition, *IL8RB *was tested because of its strong LD with 5' *SLC11A1 *markers, and found not associated with TB. Our study was the first one that tested the association of *SLC11A1 *markers with TB *and *at the same time stratified the analysis by sex or/and age. We showed that the significant association with the 3' polymorphisms of *SLC11A1 *was restricted to female patients and to young patients (age ≤65 years).

## Competing interests

The author(s) declare that they have no competing interests.

## Authors' contributions

KHL carried out nucleic acid extraction, genotyping and data analysis, and drafted the manuscript. SPY designed the study and coordinated all the activities, carried data analysis and helped to draft and finalize the manuscript. WSW, LSY, KKC, KSC, WML, EYDC, CKL, WCY recruited patients and/or control subjects, collected the required samples for the study from their respective institutes, and refined the manuscript. All authors read and approved the final manuscript.

## Pre-publication history

The pre-publication history for this paper can be accessed here:



## Supplementary Material

Additional file 1 Supplementary Table. Correction of multiple comparisons by false discovery rate (FDR) at a level of 0.05 for comparisons listed in Tables 1, 2 and 3. Click here for file
