# Supplementary material for: Sex- and age-dependent association of SLC11A1 polymorphisms with tuberculosis in Chinese: a case control study
Source: BMC Infect Dis. 2007 Mar 19;7:19. doi: 10.1186/1471-2334-7-19 (PMC1847518; doi:10.1186/1471-2334-7-19)
Supplement: Additional file 1 — Supplementary Table. Correction of multiple comparisons by false discovery rate (FDR) at a level of 0.05 for comparisons listed in Tables 1, 2 and 3. [file 1471-2334-7-19-S1.doc]

**Title:** Sex- and age-dependent association of *SLC11A1* polymorphisms with tuberculosis in Chinese: a case control study

**Corresponding author:** SP Yip

**Supplementary Table.** Correction of multiple comparisons by false discovery rate (FDR) at a level of 0.05 for comparisons listed in Tables 1, 2 and 3.^a^

| **Comparisons in Table 1** ^b^ | | |  | **Comparisons in Table 2** ^c^ | | |  | **Comparisons in Table 3** ^d^ | | |
| --- | --- | --- | --- | --- | --- | --- | --- | --- | --- | --- |
| Observed *P* values (*P_i_*) | Rank (i) | FDR thresholds  (0.05×i/6) |  | Observed *P* values, (*P_i_*) | Rank (i) | FDR thresholds  (0.05×i/8) |  | Observed *P* values (*P_i_*) | Rank (i) | FDR thresholds  (0.05×i/8) |
| **0.0163** | 1 | 0.0083 |  | **0.0031** | 1 | 0.0063 |  | 0.0158 | 1 | 0.0063 |
| **0.0165** | **2** | **0.0167** |  | **0.0047** | 2 | 0.0125 |  | 0.0408 | 2 | 0.0125 |
| 0.2096 | 3 | 0.0250 |  | **0.0049** | 3 | 0.0188 |  | 0.0419 | 3 | 0.0188 |
| 0.3438 | 4 | 0.0333 |  | **0.0075** | **4** | **0.0250** |  | 0.0450 | 4 | 0.0250 |
| 0.4780 | 5 | 0.0417 |  | 0.2370 | 5 | 0.0313 |  | 0.0478 | 5 | 0.0313 |
| 0.4957 | 6 | 0.0500 |  | 0.2876 | 6 | 0.0375 |  | 0.0798 | 6 | 0.0375 |
|  |  |  |  | 0.3023 | 7 | 0.0438 |  | 0.9276 | 7 | 0.0438 |
|  |  |  |  | 0.3479 | 8 | 0.0500 |  | 0.9668 | 8 | 0.0500 |

^a^ Each list of observed *P* values (from Tables 1, 3 and 4) is sorted from smallest [*P_1_*] to largest [*P_6_* or *P_8_*]. Each list of FDR threshold *P* values (0.05 × i/m, where i is the rank; and m is equal to 6 comparisons for Table 1, and 8 comparisons for Tables 2 and 3) is arranged from smallest to largest (0.05). Starting from the largest *P* value *P_m_*, compare *P_m_* with 0.05 × i/m. Continue as long as *P_i_* > 0.05×i/m. Let k be the first time when *P_k_* ≤ 0.05×k/m, and declare the comparisons corresponding to the smallest k *P* values as significant.

^b^ For comparisons in Table 1, k = 2 and the FDR threshold *P* value is 0.0167. Therefore, the 2 smallest observed *P* values (0.0165 and 0.0163) are significant.

^c^ For comparisons in Table 2, k = 4 and the FDR threshold *P* value is 0.0250. Therefore, the smallest 4 observed *P* values (0.0075, 0.0049, 0.0047 and 0.0031) are significant.

^d^ For comparisons in Table 3, no comparison is significant after correction by FDR.
